# Medical student simulation training in intrauterine contraception insertion and removal: an intervention to improve comfort, skill, and attitudes

**DOI:** 10.1186/s40834-016-0009-2

**Published:** 2016-02-23

**Authors:** Deborah Bartz, Amy Paris, Rie Maurer, Roxane Gardner, Natasha Johnson

**Affiliations:** 1 Brigham and Women’s Hospital, 1620 Tremont St, OBC-3, Boston, MA 02120 USA; 2 Harvard Medical School, Boston, MA USA; 3 York Hospital, York, ME USA; 4 Center for Clinical Investigation, Boston, MA USA; 5 Children’s Hospital, Boston, MA USA

**Keywords:** Intrauterine contraception, IUC, IUD, Simulation, Medical student, Medical education

## Abstract

**Background:**

Opportunities for medical students to place intrauterine contraception (IUC) in patients are rare. Our objective was to determine whether participation in an IUC insertion and removal simulation exercise would increase medical students’ comfort level with, attitudes towards, and willingness to recommend IUC.

**Methods:**

A prospective cohort study was undertaken in all students completing the obstetrics and gynecology clerkship at a major academic hospital during the 2010–2011 academic year. The exposure consisted of a 45-minute interactive didactic session and a 30-minute, hands-on practicum in IUC placement and removal using medical instruments and realistic pelvic models. Both levonorgestrel and Cu380A IUC devices were utilized. Participants completed a pre- and post-simulation survey instrument, designed to examine students’ IUC-specific knowledge, comfort, and attitudes. Pre- and post-simulation responses were compared by McNemar’s test for paired samples.

**Results:**

Thirty-five paired pre- and post-simulation surveys were analyzed, representing a 78 % response rate. Composite IUC-related knowledge scores increased by a median of 3 out of 10 points after the intervention (*p* < 0.01). Students were significantly more comfortable counseling patients about IUC as well as inserting IUC after the intervention, compared to before. Seven (20 %) students before, compared to 27 (77 %) after, agreed with the statement, “I feel comfortable placing an IUC in a patient under the supervision of an experienced doctor” (*p* < 0.01). Students developed significantly more favorable attitudes towards IUC through the intervention. Nineteen (54 %) participants before, compared to 27 (77 %) after, agreed with the statement, “I would recommend an IUC to my family member” (*p* = 0.02).

**Conclusions:**

A hands-on simulation during the obstetrics and gynecology clerkship increased medical students’ knowledge of and comfort with IUC and resulted in more favorable attitudes toward the method. Intrauterine contraception simulation in medical curricula may help expand utilization of this effective contraceptive method.

## Background

The unintended pregnancy rate in the United States is higher than in other developed countries with 51 % of the 6.6 million annual pregnancies being mistimed or undesired [1, 2]. Intrauterine Contraception (IUC) is a highly-reliable and cost-effective method of birth control with minimal side effects and a rapid return to fertility upon discontinuation [3]. Yet in 2006-10, only about 7.7 % of sexually active women in the United States had ever used IUC [4].

Underutilization of IUC is sometimes due to lack of experience on the part of clinicians and resulting lack of comfort with placement of IUCs [5]. According to multiple studies, clinicians with less experience in IUC placement are less willing to recommend IUC as a method [6–8]. Thus, increased exposure to IUC and improved training in IUC placement may represent a means to expand women’s access to this effective birth control method. Medical students, particularly those on the obstetrics and gynecology rotation, are an important group to target with improved IUC exposure, as they will go on to provide contraceptive counseling or services to patients in a variety of primary care or other medical settings.

Physicians have long been trained through the apprenticeship model summarized by the adage “See one, do one, teach one.” Medical students are taught by observing a procedure, performing one under the tutelage of a resident or attending physician, and then, in turn, teaching residents and medical students they encounter later in practice. Since learners retain more information by engaging in a task than through a passive lecture format, hands-on training has long worked well for building the medical and surgical competence of physicians-in-training [9]. However, recent changes to medicine and medical training have made patient-based training more difficult, such that alone it is insufficient to address medical students’ needs [10]. Most notably, patient safety initiatives have led to decreased duty hours that have decreased patient contact time for our students significantly [11]. Moreover, multiple learners with competing training needs often crowd the clinical field. Given the relatively low utilization of IUC, these forces limit the ability of medical students to gain hands-on experience with IUC insertion or removal.

Simulation training through the use of anatomic models can be used to bridge this procedural training gap for young physicians [11–13], and has been specifically assessed in teaching obstetric and gynecologic procedures [9, 14–16]. Jude and colleagues found that students with simulated experiences in performing vaginal deliveries expressed greater confidence in their own abilities to assist or attempt vaginal delivery in real clinical settings when compared with third-year medical students who received traditional instruction [14]. Steinauer and colleagues described a simulation activity using papayas as a uterine model to teach intrauterine procedures including endometrial biopsy, uterine aspiration, and IUC placement. The students’ knowledge of and comfort with the procedures increased from before to after the simulation [17].

This prospective cohort study seeks to assess the effect of simulation training on medical students’ comfort, confidence, attitudes, and knowledge in IUC counseling and insertion. We hypothesized that students’ comfort with IUC would improve after the intervention, and that their attitudes toward IUC would become more favorable, leading to an increased likelihood to recommend the method to appropriate patients. We also hypothesized that basic factual knowledge and ability to identify the tools used in insertion would improve.

## Methods

We conducted a prospective cohort study of all third-year Harvard Medical School students enrolled in the obstetrics and gynecology core clerkship at Brigham and Women’s Hospital throughout the 2010-2011 academic year. Enrollment in this clerkship and willingness to participate were the only eligibility requirements. All of the students rotating on the six-week clerkship--five to seven students per rotation--were approached as a group to participate. Consent was implied by reading a cover letter detailing the study and filling out the attached paper survey. Participation was voluntary and anonymous, which precluded a signed consent form since this signature would lead to identification of the participant. Harvard Medical School Institution Review Board approval was granted prior to recruitment.

**Fig. 1:**
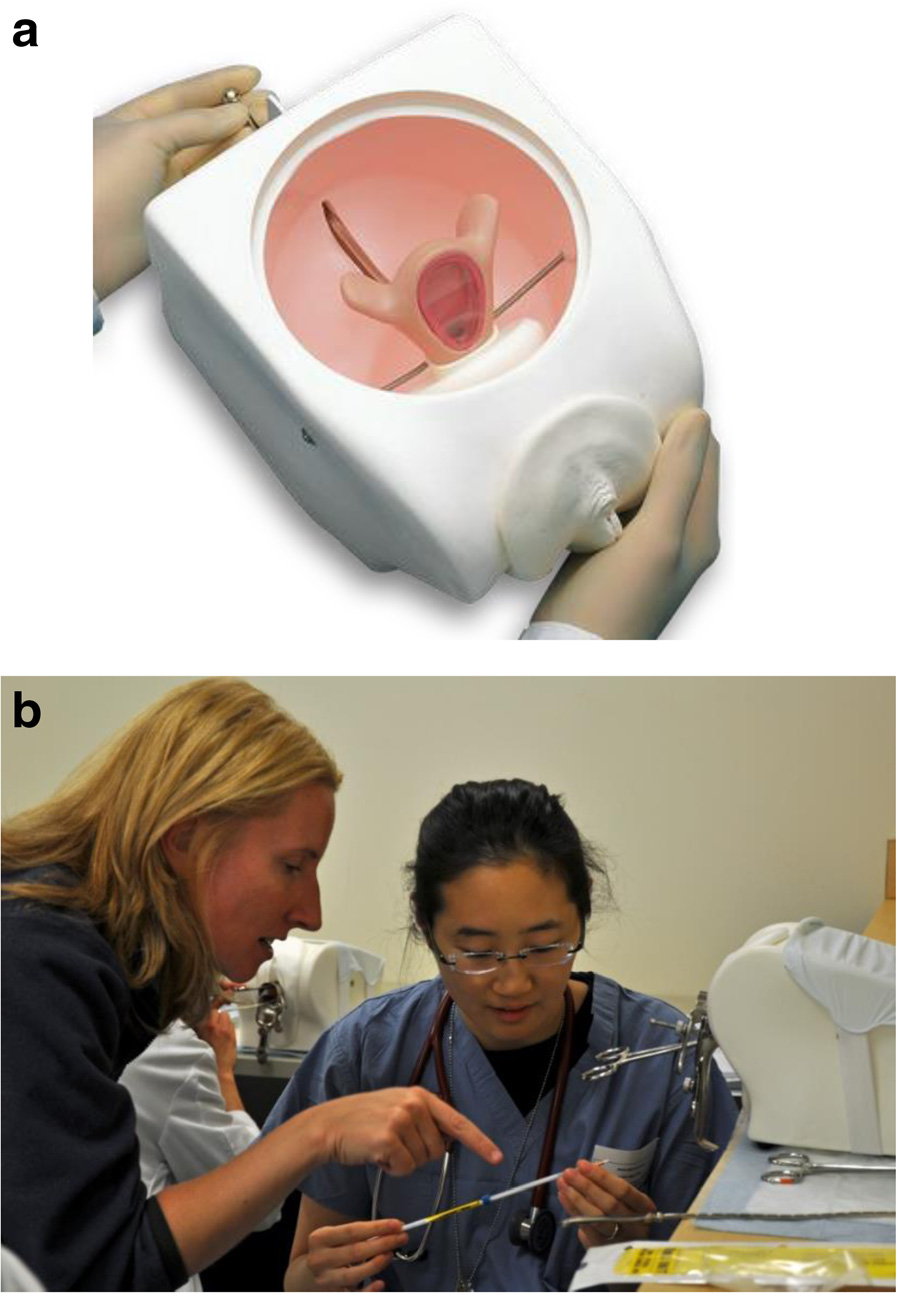
**a** Simulation pelvic model, Gaumard Scientific, Coral Gables, FL, item # S502. **b** Medical student participates in simulation activity with direct faculty teaching using simulation model, real instruments, and placebo IUC devices

The intervention of interest was a contraception didactic, which consisted of a 45-minute interactive session containing case-based teaching about IUC, followed immediately by a 30-minute, hands-on experience in IUC placement and removal using real medical tools and plastic pelvic training models (Gaumard Scientific, Coral Gables, FL, item # S502, Fig. 1a). This simulation experience consisted of a standardized demonstration in the steps of CuT 380A and levonorgestrel-releasing IUC insertion using placebo devices for these products, real instruments, and individual pelvic models (Fig. 1b). Following the demonstrations, students used the remaining time to individually practice placement of IUCs, using their own placebo devices, pelvic models, and instruments. The same faculty member gave the lecture and led the IUC simulation for all students throughout the year. Two to four faculty preceptors circulated amongst the five to seven students and provided further individual or two-on-one instruction and feedback, and were available to answer questions.

In order to assess the effectiveness of the intervention, study participants were asked to independently complete a paper survey immediately before and after the didactic and simulation experience. Pre- and post-surveys were paired by a unique, anonymous identifier. The pre-didactic survey consisted of 35 items and was meant to capture baseline IUC knowledge and attitudes that had been formed by prior IUC exposure. Participants were queried with respect to intended medical specialty, preferred learning style, and previous experience with IUC. Participants were asked questions about their interest in IUC and contraception care, as well as their comfort level with IUC counseling and insertion. Participants were also asked to rate their level of willingness to recommend IUC to appropriate patients, a proxy for assessing their attitudes toward IUC. Finally, participants’ knowledge regarding the mechanism of action and failure rate of IUC was assessed, as well as their ability to identify instruments used in the insertion procedure. The post-didactic survey included 38 items, many of which were repeated from the pre-didactic survey with regards to IUC interest and attitudes, comfort level, and knowledge.

Our primary outcome of interest was to determine whether the didactic and simulation activity had a measurable effect on students’ knowledge of, comfort level with, and attitudes towards IUC counseling and insertion. Examples of survey items assessing participants’ comfort were “I know the steps to placing an IUC”, and “I feel comfortable placing an IUC in a patient under supervision.” We asked participants to rate the degree to which they agreed with given statements on a five-point Likert scale.

Analysis was restricted to participants who completed both pre- and post-simulation surveys that could be accurately paired. Descriptive analyses were performed on the demographic data and previous IUC exposure data. Likert-scale responses were dichotomized, to either “agree” or “neutral to disagree”. The change in response from before to after the simulation was analyzed with McNemar’s test for paired samples. For knowledge-based questions, a composite score was calculated overall and for each sub-category, and the change in score from before to after the intervention was compared using the Signed Rank Test, as data were not normally distributed.

## Results

All 45 Harvard Medical School students completing their obstetrics and gynecology clerkships at Brigham and Women’s Hospital during the 2010–2011 academic year were eligible and invited to participate. Of these, 35 returned paired surveys, for a response rate of 78 %.

When asked what medical specialty participants planned to pursue, eleven responded that they planned to go into surgery, two into obstetrics and gynecology, eight into primary care subspecialties, and three into medicine subspecialties; nine participants were undecided. More than half of participants (54 %) had learned about IUC in a previous medical school lecture; however, only a minority had firsthand experience with IUC, with nine (26 %) who had seen at least one IUC placed and ten (29 %) who reported personal IUC use or exposure through use of a family member, friend, or partner. With regards to learning style, 17 respondents (49 %) chose “participating in hands-on activities” as the way they learned best, compared to “reading about a topic on my own” (31 %), “teaching someone else about a topic” (11 %), “attending a lecture on the topic” (5.7 %), or “participating in a group discussion” (2.9 %) (Table 1).

**Table 1:** Baseline characteristics of all study participants (*N* = 35)

Characteristic	Category	*n* (%)
Planned Specialty	Surgery	11 (31.4)
	Medicine	5 (14.3)
	Pediatrics	3 (8.6)
	OB-GYN	2 (5.7)
	Undecided	9 (25.7)
	Other No answer	3 (8.6) 2 (5.7)
Prior Experience with IUC	Learned about IUC in a previous med school lecture	19 (54.3)
	Previously seen at least one IUC placement	9 (25.7)
	Self, partner, or family member has used IUC	10 (28.6)
Student learns best by:	Participating in hands-on activities	17 (48.6)
	Reading about a topic on my own	11 (31.4)
	Teaching someone else about a topic	4 (11.4)
	Attending a lecture on a topic	2 (5.7)
	Participating in a group discussion	1 (2.9)

Students improved their scores on knowledge-based questions after the simulation, compared to before. Of the three questions that tested knowledge of IUC mechanism of action and failure rates, the mean answers correct increased from 1.34 before to 2.06 after (*p* < 0.01). Of seven questions that asked students to identify instruments used in IUC insertion, the median number of correct responses increased 2.0 points, from 5.0 to 7.0 (*p* < 0.01).

Students became more comfortable with IUC after the simulation, compared to before, as evidenced by significantly more positive responses to all five IUC comfort questions. For example, 7 (20 %) students before, compared to 27 (77 %) after, replied that they would feel comfortable placing an IUC in a patient under the supervision of an experienced doctor (*p* < 0.01). Ten (29 %) students before the intervention compared to 24 (69 %) after reported comfort with counseling patients about the IUC (*p* < 0.01) (Table 2).

**Table 2:** Responses to IUC comfort-related items

Statement	Agree before *n* (%)	Agree after *n* (%)	*P* values
I am able to counsel patients about the IUC	10 (29)	24 (69)	<0.01
I know the steps to placing an IUC	5 (14)	31 (89)	<0.01
I feel comfortable placing an IUC independently in a plastic pelvic model	5 (14)	32 (91)	<0.01
I could teach another student how to place an IUC in a plastic pelvic model	0 (0)	28 (80)	NA
I feel comfortable placing an IUC in a patient under the supervision of an experienced doctor	7 (20)	27 (77)	<0.01

Participants were also more likely to recommend IUC to appropriate patients after the intervention. In all four cases where IUC was an appropriate method, more students after compared to before the intervention were willing to recommend IUC; two out of four of these achieved statistical significance (Table 3). In addition, after the intervention, 27 (77 %) of participants “agreed” or “strongly agreed” with the statement, “I would recommend an IUC to my family member,” compared to 19 (54 %) before (*p* = 0.02).

**Table 3:** Responses to IUC attitude-related items

Statement: assuming she was otherwise a good candidate, I would recommend an IUC for a patient:	Agree before *n* (%)	Agree after *n* (%)	*P* values
Who has never been pregnant	20 (59)	25 (74)	0.17
Who is under 19 years old	15 (43)	27 (77)	<0.01
Who has multiple partners	11 (33)	16 (48)	0.06
Who has had more than one vaginal delivery	20 (57)	30 (86)	<0.01

Overall, there was a high level of interest in IUC both before and after the intervention. Thirty-two (91 %) students reported wanting to learn more about IUCs prior to the simulation, compared to 30 (86 %) after (*p* = NS). There was a trend toward more students indicating that contraceptive counseling would be a part of their practice after the simulation 25 (71 %), compared to before 21 (60 %) (*p* = NS).

## Discussion

Intrauterine contraception is an underutilized method in the United States, and prior studies have indicated that physician lack of experience with IUC and resulting lack of comfort are partially responsible for an unwillingness to recommend the method [5–8]. Our study demonstrates that simulation of IUC insertion and removal as part of the medical student obstetrics and gynecology clerkship is a successful means to improve comfort with and attitudes towards IUC in student physicians who otherwise may not gain hands-on experience with this contraceptive method before graduation. Participants became more adept at recognizing appropriate candidates for IUC after the intervention, and they became significantly more likely to recommend IUC to a friend or family member. Students planning to go into a wide variety of specialties reported a high level of interest in IUC both before and after the intervention, and there was a trend toward more students planning to incorporate IUC counseling into their medical practice.

The underutilization of IUC by women in the U.S. has been shown to have a negative impact on physicians in training. A survey of graduating U.S. obstetrics and gynecology residents was performed in 1992, when IUC use was at its nadir in this country. The survey was administered 6 months prior to graduation and revealed that 38 % of chief residents had never placed an IUC, 71 % had not placed more than 10 IUCs, and 25 % had never received any instruction on IUC insertion during their residency [18]. Since that time, the percentage of U.S. women currently using the IUC has increased from 0.5 % in 1995 to 5.5 % in 2008 [19]. However, opportunities to learn about IUC in medical school continue to be infrequent, with only 54 % of third year medical students in our study reporting having learned about IUC in a previous medical school lecture and only 26 % reporting having ever seen an IUC placed prior to their first week of the obstetrics and gynecology clerkship. In a U.S. and Canadian survey of all clerkship coordinators, Steinauer and colleagues found that only 77 % of U.S. and Canadian medical schools report preclinical education on intrauterine contraception [20]. Therefore, simulation-based IUC training has great potential to improve the obstetrics and gynecology clerkship curriculum; even if it is the only exposure students get to hands-on IUC insertion. Our results agree with Khadivzadeh and colleagues who found that midwifery students that participated in simulation-based training of IUC insertion had significantly lower levels of anxiety in managing real patients compared to those midwifery students who participated in traditional training of IUC insertion [21].

Conducted over the course of a full academic year, this study is an ambitious evaluation of a medical education intervention. We obtained a high response rate and collected detailed information to assess several parameters of educational impact of our intervention. There are several limitations to this study. Notably, the involvement of only a single educational institution and resulting small sample size may limit the ability to generalize our results to other student populations. Second, because the survey was given before both the didactic session and the simulation, it is impossible to isolate the effects of the simulation from those of the didactic. Simulation-based educational activities are often preceded by content-based lectures. This curricular design with a simulation preceded by a didactic may even allow for the greatest educational impact to be gained from the simulation itself. Cook and colleagues conducted a systematic review of technology enhanced simulation training for health professions learners compared to training that did not use technology enhanced simulation [22]. They found that technology enhanced simulation training for health professions education was consistently associated with large effects for knowledge, skills and behaviors and moderate effects for patient-related outcomes compared to training without use of such technology. Third, it is unclear whether the gain in skill and confidence seen after our simulation will translate into actual patient encounters. In other studies of simulation-based educational activities in the field of obstetrics and gynecology, greater procedure-based exposure has translated to improved clinical performance [9]. Lastly, while participants felt more comfortable immediately after practicing IUC insertion, further study is needed to determine whether the observed increase in comfort and improved willingness to recommend IUC will be sustained over the long-term. Prospective follow-up is needed to ascertain long-term effects of our intervention. Other areas for future inquiry could include targeting populations of trainees who are more likely to provide primary care women’s health, such as primary care residents or nurse midwifery students, to see if they would similarly benefit from this simulation training.

## Conclusion

This study corroborates the one previous published study demonstrating that a simulation-based didactic on IUC counseling and insertion improves participants’ comfort levels with IUC. Our study further demonstrates that the participation in the simulation is associated with an increased willingness to recommend this underutilized method. Our results support the incorporation of this didactic activity into the standard curriculum of the third year obstetrics and gynecology clerkship.

## Abbreviation

IUC: intrauterine contraception
